# Toward facilitating microalgae cope with effluent from anaerobic digestion of kitchen waste: the art of agricultural phytohormones

**DOI:** 10.1186/s13068-017-0759-3

**Published:** 2017-03-24

**Authors:** Haiyan Pei, Liqun Jiang, Qingjie Hou, Ze Yu

**Affiliations:** 1 School of Environmental Science and Engineering, Shandong University, No. 27 Shanda Nan Road, Jinan, 250100 China; 2 Shandong Provincial Engineering Centre on Environmental Science and Technology, No. 17923 Jingshi Road, Jinan, 250061 China

**Keywords:** Agricultural phytohormones, Anaerobic digestion, Kitchen waste, Algae biomass

## Abstract

**Background:**

Although numerous studies have used wastewater as substitutes to cultivate microalgae, most of them obtained weaker algal viability than standard media. Some studies demonstrated a promotion of phytohormones on algal growth in standard media. For exploiting a strategy to improve algal biomass accumulation in effluent from anaerobic digestion of kitchen waste (ADE-KW), the agricultural phytohormones gibberellin, indole-3-acetic acid, and brassinolide (GIB) were applied to *Chlorella* SDEC-11 and *Scenedesmus* SDEC-13 at different stages of algal growth. Previous studies have demonstrated a promotion of phytohormones on algal growth in standard media, but attempts have been scarce, focusing on wastewater cultivation system. In addition, the effects of wastewater on algal morphology and ultrastructure have not been revealed so far, much less on the mechanism of the role of phytohormones on algae.

**Results:**

ADE-KW disrupted the membranes of nuclear and chloroplast in ultrastructural cell of SDEC-11, and reduced the room between chloroplast and cell membrane and increased the starch size of SDEC-13. This reduced algal growth and biocompound accumulation, but SDEC-13 had greater adaptation to ADE-KW than SDEC-11. Moreover, inoculation with an algal seed pretreated with GIB aided the adaptability and viability of algae in ADE-KW, which for SDEC-13 was even promoted to the level in BG11. GIB mitigated the inhibition of ADE-KW on algal cell division and photosynthetic pigments and apparatus, and increased lipid droplets, which might result from the change in the synthesis and the fate of nicotinamide adenine dinucleotide phosphate. GIB addition significantly promoted lipid productivity of the two algal species, following 13 mg L^−1^ d^−1^ of SDEC-11 in B^+^ADE-KW and especially 13 mg L^−1^ d^−1^ of SDEC-13 achieved during the priming of algal seed with the hormones, which is 139% higher than 5 mg L^−1^ d^−1^ achieved in ADE-KW control.

**Conclusions:**

Agricultural phytohormones could be applied as a strategy for promoting biomass and biocompound accumulation of algae in ADE-KW, in which pretreatment of the algal inoculum with hormones is a unique way to help algae survive under stress. Considering our results and treatment technology for kitchen waste, a more feasible and economic plant can be built incorporating anaerobic digestion, algae cultivation with ADE-KW assisted with phytohormones, and biodiesel production.

**Electronic supplementary material:**

The online version of this article (doi:10.1186/s13068-017-0759-3) contains supplementary material, which is available to authorized users.

## Background

To make microalgae-based biofuel practically viable, combining wastewater and algae biomass cultivation has been regarded as an economic and environmentally sustainable approach by many researchers. The combination could ease the bottleneck of high cost in nutrient demand and vast water input required for simultaneous algae growth and wastewater treatment [1–4]. For several kinds of wastewater that have been reported to be feasible alternatives for algae cultivation, the biomasses or lipid productivities decreased compared to the levels achieved in standard medium. Therefore, some steps must be taken to facilitate algae to perform in wastewater as well as, or if possible better than, they do in their favorable artificial media, prior to large-scale production [5]. A strategy must be adopted, which supports microalgae to maximize their potential to survive in nonideal environment.

As regards the wastewater in this study, we chose the effluent from anaerobic digestion of kitchen waste for the following reasons. Kitchen waste is generated daily all over the world and accounts for a large part of municipal waste [6]: for example, approximately 60 billion kilograms of food supply are wasted in the United States [7]; it contributes up to 60% of the total municipal solid waste in Egypt [8]; more than 60 billion kg of kitchen waste are produced in China every year. Anaerobic digestion is an effective and common way to deal with kitchen waste, as it can reduce biodegradable waste and produce H_2_ or biogas, and hence, the renewable energy too [9]. However, in the process of anaerobic digestion, the vast amount of residual effluent produced becomes another problem due to the presence of luxuriously rich nutrients in it. Due to the gradually maturing and advanced technology of anaerobic digestion for kitchen waste, the large amount of effluents generated in this process could be chosen for cultivating microalgae to produce biomass along with nutrient removal from wastewater. Moreover, infrastructure for biodiesel production usually exists as a component of kitchen waste treatment, taking advantage of oil separated from the waste, which might be employed to produce algae-based biofuel. Meanwhile, the existing processes for anaerobic digestion are also important for microalgae-based biodiesel sustainability, which can solve the issues of algal residue after oil extraction and energy balance [10].

There have been a few applications of using effluent from anaerobic digestion processes, with the digester feedstocks including livestock waste [11], municipal wastewater [2], and food wastewater [12]. However, so far, limited research has been reported on using effluent from anaerobic digestion of kitchen waste (ADE-KW) to cultivate algae. There are differences between the effluents from the digestion of kitchen waste and those from livestock waste, municipal, and food wastewaters, especially in terms of dark color, high organic matter levels, and limited phosphorus concentration, as shown in Additional file 1: Table S1 [2, 11–15]. The imbalance in nutrient ratios and the dark color pose challenges to algal growth. It is necessary to confirm whether it is feasible to use ADE-KW to cultivate algae or employ algae to develop a process for nutrient removal from the wastewater.

In terms of increasing biomass and lipid productivity, phytohormones are taken into consideration due to their role in plant growth regulation [16, 17]. Their use has been extended to the field of algae production [18–22]. Plant hormones are growth regulators, and at an appropriate concentration, they appear to play a regulatory role in microalgae cell development including cell division or elongation, and in chlorophyll and protein metabolisms [21–23]. Besides promoting growth, phytohormones also have been reported to enhance tolerance to stress, such as heavy metals [20, 24], oxidative conditions [25], and osmotic and salt stresses [26]. Nevertheless, the hormones applied heretofore in algal cultivation were pure chemicals, which accounted for the high cost of production and addition required after starting batch cultivation.

The hormones commonly applied in agriculture, i.e., agricultural phytohormones, are characterized by mixed material containing more than one kind of phytohormone, and a mature production line with low investment for large-scale production. However, the application of agricultural phytohormones to microalgae had not been studied. Furthermore, the previous studies did not mention the impact of phytohormones on algal activity in response to a complex stress environment such as real wastewater, except in applying hormones to help algae deal with a simple unfavorable factor [20, 24]. Therefore, the main aim of this study was to demonstrate whether the agricultural phytohormones could enhance the algae’s ability to tolerate the adverse effect of wastewater and achieve a considerable amount of biomass and accumulation of biocompounds.

Inspired by the approach of applying phytohormone to higher plants, we tried applying it in seed priming and during inoculum preparation, as the former enhances the activity of higher plants under stress [27], while the latter pretreats the algal seed before batch cultivation. This study enables us to understand the influence of effluent from anaerobic digestion of kitchen waste (ADE-KW) on algae growth and biocompounds accumulation, and further verify the potential of using phytohormones to help algae overcome the adverse effects from the wastewater feedstock and provide an economical way to accumulate algae biomass based on ADE-KW as media.

## Methods

### Materials

The effluent from anaerobic digestion of kitchen waste (ADE-KW) was collected from Shandong Shifang Environmental Protection & Bio-Energy Company (Jinan, PR China). The digestion process operates continuously in the company and ultimately produces consistent effluent, with a relative deviation in water characteristics of less than 10% between different batches. Following our previous survey on the effect of ADE-KW loading on algal growth (data not yet published), ADE-KW diluted by a factor of 15 with tap water was put into use as the growth medium for the tested algae, without employing any other pretreatments such as filtering, autoclaving, or pH adjustment. The chemical composition of the 15-fold diluted ADE-KW was as follows: TN 138.22 ± 0.72 mg L^−1^, NH_3_-N 105.41 ± 0.73 mg L^−1^, TP 1.75 ± 0.07 mg L^−1^, COD_Cr_ 390.16 ± 2.32 mg L^−1^, protein 33.52 ± 2.23 mg L^−1^, carbohydrates 11.09 ± 2.78 mg L^−1^, pH 8.54 ± 0.05.

The tested microalgae *Chlorella ellipsoidea* (SDEC-11) and *Scenedesmus quadricauda* (SDEC-13) were similar to those used by Jiang et al. [22], which were isolated and evaluated as the good candidates for biofuel production by our laboratory. *Chlorella* and *Scenedesmus* have been demonstrated as ideal candidates for biofuel industrialization due to their strong adaptation to wastewater, inhibition of bacterial growth, and relatively high lipid production [28, 29]. Algae inoculums were prepared from exponentially grown seed cultures in BG11 medium which contains 1.5 g L^−1^ NaNO_3_, 40 mg L^−1^ K_2_HPO_4_, 75 mg L^−1^ MgSO_4_ 7H_2_O, 36 mg L^−1^ CaCl_2_·2H_2_O, 6 mg L^−1^ citric acid, 6 mg L^−1^ ferric ammonium citrate, 1 mg L^−1^ EDTA-Na_2_, 20 mg L^−1^ Na_2_CO_3_, and 1 mL L^−1^ A_5_. A_5_ is a trace metal solution containing 2.86 g L^−1^ H_3_BO_3_, 1.86 g L^−1^ MnCl_2_·4H_2_O, 0.22 g L^−1^ ZnSO_4_·7H_2_O, 0.39 g L^−1^ Na_2_MoO_4_·2H_2_O, 0.08 g L^−1^ CuSO_4_·5H_2_O, and 0.05 g L^−1^ Co(NO_3_)_2_·6H_2_O. If there is no special statement, the chemicals are produced by Sinopharm.

The phytohormones (VitaCat, Germany) were commonly applied in agriculture, and consisted of 0.135% (w/w) gibberellin (GA_3_), 0.00052% (w/w) indole-3-acetic acid (IAA), 0.00031% (w/w) brassinolide (BL), and an auxiliary ingredient that helps the former three phytohormones to become powder that can easily dissolve in water, called GIB for short.

### Experimental set-up

GIB phytohormones were incorporated into the medium at 3 days before inoculation during the preparation phase of the algae inoculum seed (S^+^) and at the beginning of the batch cultivation phase of the algae (B^+^). The priming seed for S^+^ group was prepared with these procedures: (1) cultivating tested algae to reaching the middle exponential phase; (2) adding the phytohormones to culture mentioned at last step; (3) 3 days later, recovering the algae by centrifugation and washed three times with deionized water; (4) inoculating the pellet from the third step into the wastewater for S^+^ADE-KW run. The seed for B^+^ADE-KW group was prepared according to abovementioned process in absence of the second procedure. Cultivation in BG11 and ADE-KW with no GIB addition was conducted simultaneously as controls for all experiments. The overall experimental set-up is presented in Table 1 and run names suggest the phase at which hormones were added and the culture medium (e.g., B^+^ADE-KW indicates that phytohormones were added to algae cultured with ADE-KW at the batch cultivation phase).

**Table 1:** Experimental set-up, maximum biomass concentration, and average biomass productivities of *Chlorella* SDEC-11 and *Scenedesmus* SDEC-13

Run^e^	Phytohormones dose (mg L^−1^)				Maximum biomass concentration (g L^−1^)		Verhulst model			
	Seed preparation		Batch cultivation				*X*_max_ (g L^−1^)		*µ* (d^−1^)	
	SDEC-11	SDEC-13	SDEC-11	SDEC-13	SDEC-11	SDEC-13*	SDEC-11	SDEC-13	SDEC-11	SDEC-13
BG11	–	–	–	–	0.61 ± 0.01^a^	0.47 ± 0.04^a^	0.70 ± 0.05	0.70 ± 0.17	0.31 ± 0.04	0.17 ± 0.02
ADE-KW	–	–	–	–	0.30 ± 0.03^b^	0.22 ± 0.01^b^	0.25 ± 0.01	0.21 ± 0.01	0.12 ± 0.06	0.18 ± 0.08
S^+^ADE-KW	10	50	–	–	0.31 ± 0.01^bc^	0.40 ± 0.01^c^	0.32 ± 0.04	0.38 ± 0.05	0.16 ± 0.06	0.27 ± 0.10
B^+^ADE-KW	–	–	10	50	0.33 ± 0.01^c^	0.33 ± 0.01^d^	0.34 ± 0.03	0.35 ± 0.03	0.18 ± 0.05	0.15 ± 0.04

^e^Run names indicate the phase at which hormones were added and the culture medium, e.g., B^+^ADE-KW indicates that phytohormones were added to algae cultured with ADE-KW at the batch cultivation phase. – Phytohormone not added. Data are the means of three independent experiments ± SD

* Data in the same column followed by different letters are significantly different by Duncan’s test at *p* < 0.05

GA_3_ is the main hormone in the phytohormones, so the GIB dose was calculated according to the previous research by Tatkowska and Buczek [30] and Tate et al. [31], to provide GA_3_ at an appropriate concentration in culture medium (about 1 × 10^−7^ M for *Chlorella* sp. and 5 × 10^−7^ M for *Scenedesmus* sp.). Erlenmeyer flasks (1 L) containing 800 mL culture sealed with 8-layer gauze and *Parafilm* were used as the batch reactors. All of these bioreactors were placed on a shaker table with the following cultivation conditions: photosynthetic photon flux of 30 μmol m^−2^ s^−1^ provided by daylight fluorescent tubes (Philips, 36 W), temperature 25 ± 3 °C and continuous shaking (120 rpm). The light intensity was detected by an irradiance sensor (ZDS-10, Shanghai Cany Precision Instrument, China). All the experiments were conducted in triplicate.

### Analytical procedures

#### Determination of algal growth

Algal growth was evaluated every 48 h by the increase of microalgae biomass concentration, measured through dry weight (g L^−1^) as Jiang et al. described, and the cell number in the bioreactors [3]. The cell number was determined by direct counting of cells in the growth medium using a blood counting chamber under a microscope (CX31, Olympus, Japan).

For simulating algae growth, the Verhulst logistic kinetic model was used as the previous report did [32] and the equation was shown below:

1 $$X = \frac{{X_{{\rm max} } }}{{1 + e^{{m} - {\mu t}} }}$$

where *X* is the concentration of dry algal biomass (g L^−1^); *X*_max_ is the maximum algae biomass concentration (g L^−1^); *m* is a constant in the logistic model indicating the relative position from the origin; *μ* is the specific growth rate (d^−1^); and *t* represents the culture time (*d*).

The investment of algae biomass production from different culture patterns was calculated in the context: (1) the phytohormones was purchase at a price of 2.4 CNY g^−1^; (2) the total chemicals providing nutrient in 1 m^3^ BG11 medium cost 85.31 CNY according to our purchase record.

#### Determination of photosynthetic pigments, lipid, carbohydrate, and protein

Detection and calculation of total pigment content was carried out as Pancha et al. reported [33]. The process include: every 48 h, 1.5 mL of algal culture was taken into a 2 mL plastic centrifuge tube and centrifuged at 10,000 rpm for 5 min; the supernatant was discarded and 1.5 mL of 99.9% methanol was added to the pellet, mixed well and incubated at 45 °C for 24 h in darkness; after incubation, the pigment content was determined on a UV–vis spectrophotometer (UV-2450, Shimadzu, Japan).

The analyses of lipid, carbohydrate, and protein in biomass were conducted as per the procedure in the previous study: (1) harvest of biomass at the early stationary stage by centrifugation at 4000 rpm for 10 min; (2) drying of the algae pellet to constant weight at −50 °C in a lyophilizer (EYELA FDU-1200, Tokyo Rikakikai Co., Japan), and then grinding to a homogeneous powder; (3) quantification of total crude lipid gravimetrically in dry algal biomass after extracting with chloroform and methanol, and calculating the average lipid productivity; (4) summary of the total protein content through amino acid composition; (5) determination of carbohydrate’s content colorimetrically with a Multiskan device (Multiskan FC, Thermo, USA); and (6) examination of fatty acid (FA) properties through GC-MS [3].

#### Determination of cell size

At the stationary stage, observation of algal cell size was performed using an inverted fluorescence microscope (Ti-s, Nikon, Japan), and statistical analysis of the semi-major axis and semi-minor axis of algae cells was then implemented with NIS-Elements D 4.20.00 software. A count of cells undergoing division and the number of daughter cells retained in a single cell before release was conducted with the microscope (CX31, Olympus, Japan), judging by the number of autospores in one cell.

#### Determination of water characteristics

After algae biomass was harvested through centrifugation, the suspension was filtered and the filtrate was used for testing COD_Cr_, NH_3_-N, TN and TP concentrations following Chinese state standard testing methods [34]. In detail, COD_Cr_ was measured using the potassium dichromate method; NH_3_-N, TN and TP concentrations were determined according to the Nessler’s reagent spectrophotometric method (PRC National Standard, HJ 535-2009), alkaline potassium persulphate oxidation-ultraviolet spectrophotometry (GB 11894-89), and ammonium molybdate spectrophotometric method (PRC National Standard, GB 11893-89), respectively.

#### Transmission electron morphology

Algae taken from the logarithmic phase was made into block mass of 1–3 mm^3^, then they were promptly fixed at 4 °C in 2.5% glutaraldehyde prepared for 4 h and subsequently post-fixed in a 1% osmium-tetroxide in phosphate buffer for 1–1.5 h. Next, the samples were dehydrated in a graded acetone series from 50% to absolute acetone, embedded in Epon-812 (SPI-Chem). Sections were cut with a diamond knife (2.1 mm, Diatome, Switzerland) and placed on Formvar (F6146, Sigma) and carbon-coated copper grids (T110215, Beijing Xinxing Braim Technology, China), and then stained with uranyl acetate and lead citrate for each 15 min, and examined under a JEM-1200EX transmission electron microscope (JEOL, Akishima, Tokyo, Japan).

### Statistical analysis

Results are presented in the form of mean value ± standard deviation from three independent experiments. The differences in biomass production, cell division and content of photosynthetic pigments, lipid, carbohydrate and protein between experimental groups were analyzed using one-way analysis of variance (ANOVA). A difference was considered statistically significant when *p* < 0.05.

## Results and discussion

### ADE-KW adverse effect on the growth potential of algae

**Fig. 1:**
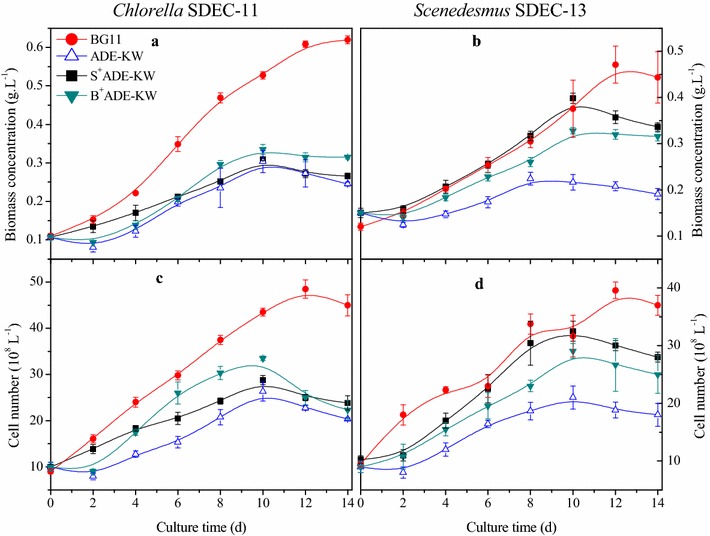
The effect of phytohormones on biomass concentration (**a**, **b**) and cell density (**c**, **d**) of *Chlorella* SDEC-11 and *Scenedesmus* SDEC-13 in ADE-KW. The run names in the legend indicate the phase at which hormones were added and the culture medium; e.g., B^+^ADE-KW indicates that phytohormones were added to algae cultured with ADE-KW at the batch cultivation phase

**Fig. 2:**
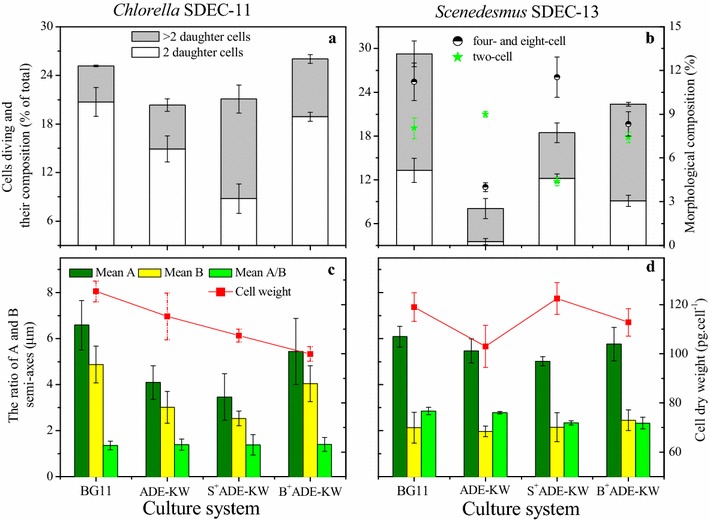
The effect of phytohormones on cell division (**a**, **b**) and morphology (**c**, **d**) of *Chlorella* SDEC-11 and *Scenedesmus* SDEC-13. In **c** and **d**, A: semi-major axis; B: semi-minor axis; A/B: the ratio of A and B axes. The run names on the abscissa indicate the phase at which hormones were added and the culture medium; e.g., B^+^ADE-KW indicates that phytohormones were added to algae cultured with ADE-KW at the batch cultivation phase

As shown in Fig. 1, algae grew slower in ADE-KW than in BG11, and a 2-day lag phase was observed in the wastewater. In ADE-KW, the decreased cell number, along with dramatic inhibition cells undergoing division (*p* < 0.05) as shown in Fig. 2, also indicated weaker growth than in BG11. However, the wastewater reduced the biomass of *Chlorella* SDEC-11 by about 50%, while *Scenedesmus* SDEC-13 in ADE-KW obtained a biomass concentration of 0.22 g L^−1^, equivalent to 70% of the figure in BG11.

**Fig. 3:**
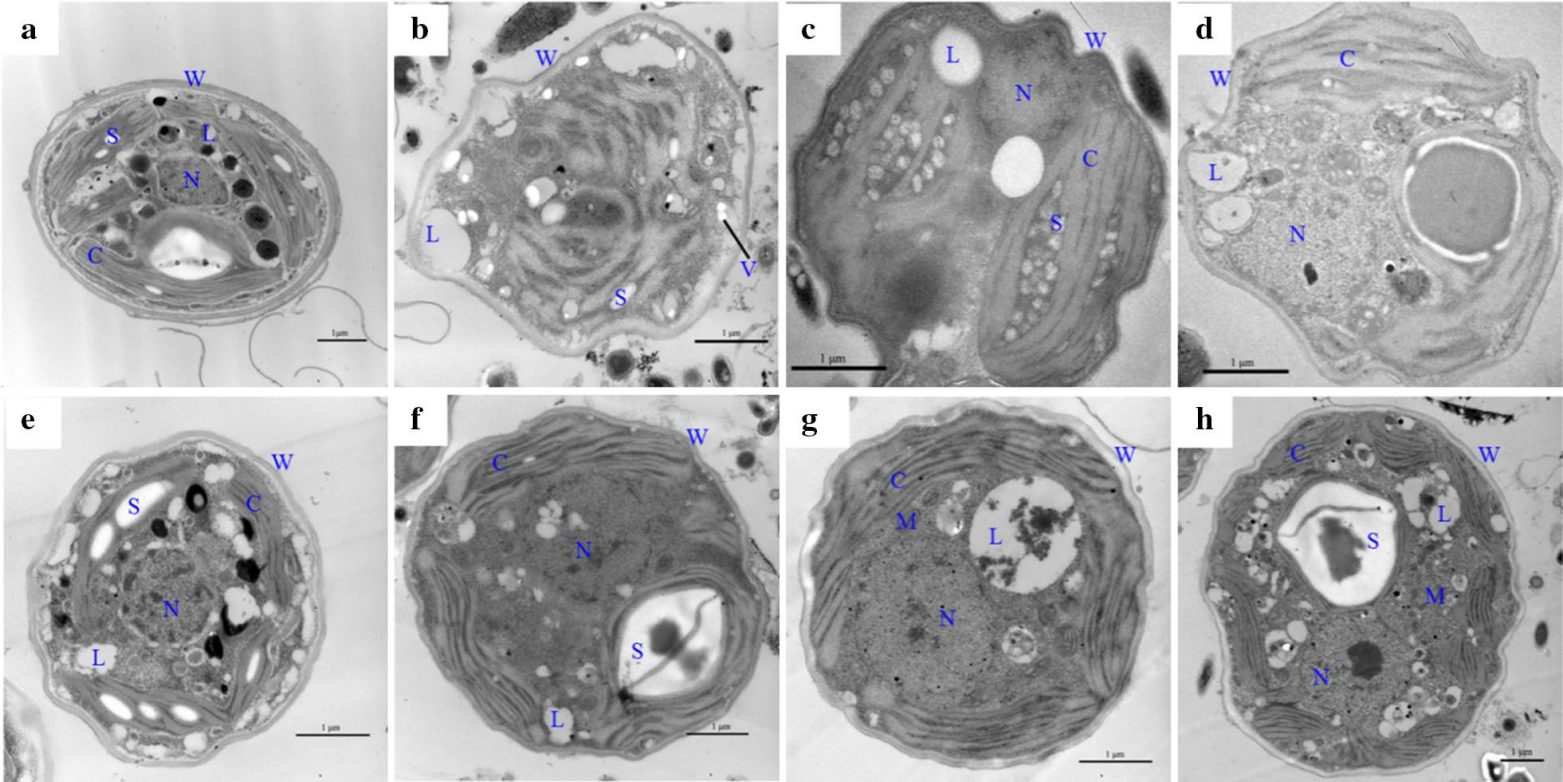
Details of ultrastructure of *Chlorella* SDEC-11 and *Scenedesmus* SDEC-13 treated with ADE-KW and phytohormones. **a** SDEC-11 in BG11; **b** SDEC-11 in ADE-KW; **c** SDEC-11 in S^+^ADE-KW; **d** SDEC-11 in B^+^ADE-KW; **e** SDEC-13 in BG11; **f** SDEC-13 in ADE-KW; **g** SDEC-13 in S^+^ADE-KW; **h** SDEC-13 in B^+^ADE-KW. *C* chlorophyll, *S* starch, *L* lipid, *W* cell wall, *N* cell nucleus, *M* mitochondrion, *V* vacuole. The above run names indicate the phase at which hormones were added, and the culture medium; e.g., B^+^ADE-KW, indicates that phytohormones were added to algae cultured with ADE-KW at the batch cultivation phase. *Scale bar* 1 μm

Under an electron microscopy, the anomalies in ultrastructural images verified that the ADE-KW was not an ideal environment for the growth of *Chlorella* SDEC-11 (Fig. 3). From the ultrastructural point of view, SDEC-11 cells in control culture showed intact oval shape and the typical organelles including a nucleus coated by a nuclear envelope, parietal chloroplasts distributed around cell membrane (Fig. 3a). Compared to normal culture, cells in ADE-KW presented radical changes including damage in the cell wall, disappeared organelle membrane and appearance of vacuoles. Damage in the cell wall was presented by the three layers participated in the formation of crests. A totally disruption of all organelles in the cytoplasm was observed that no nucleus, chloroplast or even clear thylakoid appeared and incompact reticular thylakoids covered the whole cell (Fig. 3b). In the cytoplasm of most cells, vacuoles were also observed for cellular detoxification mechanism because vacuoles act as an ion storage or compartmentalization of toxic materials [35].

Only slight change caused by the wastewater was observed for SDEC-13, where obvious nucleus and chloroplast existed in algal cell cultured with ADE-KW, as Fig. 3f shown. With respect to *Scenedesmus* cell of control, well-developed organelles and sporadic starch granules in chloroplast were observed (Fig. 3e); in contrast, after culturing with ADE-KW, starch granule in cell presented in a big one and the room between chloroplast and cell membrane became smaller. However, nucleus, chloroplast and mitochondrion still maintained their complete shape. These results indicated a greater adaptation of *Scenedesmus* SDEC-13 than *Chlorella* SDEC-11.

The main reasons for poor growth of microalgae in the real wastewater might be the extreme imbalance in nutrition (N/P: 78.95), and the inevitable cross-infection or contamination of bacteria observed under the microscope. Bacterial infection had become a big constraint in mass cultivation and impeded the industrial process in other research, through impacting microalgae with either direct or indirect attack which might destroy the structure of DNA or restrain photosynthesis of certain microalgae strains [36]. As the ultrastructure in Fig. 3b, f shown, the bacteria assuredly survived in culture system prepared with the wastewater. Besides the two factors in ADE-KW inhibiting algal survival, a decrease growth rate of algae also resulted from the color and particles suspended in the wastewater, visually as dark color, since they could absorb, scatter or interference the light and ultimately reduce photosynthetic active radiation for chlorophyll utilization.

Microalgae employ chlorophyll for light absorption to provide primary energy for all activities in the cell. However, the unfavorable environment of ADE-KW led to a decrease in all photosynthetic pigments of *Chlorella* SDEC-11, with losses in chlorophyll (chl-) a and b contents by about 73%, which were also expressed as a change in the color of a culture from green to yellow or light yellow and incomplete chloroplast shown in ultrastructure. Cultivation of *Scenedesmus* SDEC-13 with ADE-KW also showed 44 and 48% reductions in chl-a and chl-b, respectively. Moreover, the dark color and suspended solids in the wastewater also decreased the light quality reaching the algal cells. Increased ratios in chl-a/chl-b have been considered to decrease light collection in relation to the rate of photosystem II (PS II) photochemistry, while increments in carotenoids/chl-a + b (caro/chl-a + b) indicated the difficulty in light harvesting complex and PS II activity [37]. For the two algae, the ratio of chl-a/chl-b and caro/chl-a + b increased dramatically in ADE-KW media compared with BG11 culture (*p* < 0.05), which indicated abnormal chloroplast photosynthetic phosphorylation activity and acclimation of chlorophyll when subjected to the harsh condition.

The ADE-KW wastewater decelerated the algal growth including biomass accumulation and cell density, which might occur through slowing down of the rate of cell division and chlorophyll activity. In terms of the degree of decline in the above metrics, *Scenedesmus* SDEC-13 has a stronger adaptability to ADE-KW than *Chlorella* SDEC-11.

### Role of phytohormones in stimulating algae vitality in ADE-KW

The effect of the GIB phytohormones on the growth of algae cultivated in ADE-KW, expressed as biomass concentration and cell number, is presented in Fig. 1. Hormone addition during the seed preparation phase or the batch cultivation phase increased biomass accumulation slightly for *Chlorella* SDEC-11, but with a statistical difference for *Scenedesmus* SDEC-13 (*p* < 0.05), compared with nontreated ADE-KW control (Table 1). Ozioko et al. also found no significant effects of GA_3_ on cell growth of *Chlorella* sp. IAM-C212, as expressed by dry cell concentration [38]. However, this pretreatment of the seed with phytohormones caused the two algae strains to have no lag phase when they encountered ADE-KW, and especially gave SDEC-13 stronger bioactivity to survive in ADE-KW, attaining 0.40 g L^−1^ biomass, nearly double that of 0.22 g L^−1^ obtained in the ADE-KW control.

Conformance between the empirical results and simulation from the Verhulst model was observed (Table 1). No significant difference appeared for biomass production of the two algae strains under the condition of GIB hormones. Nor was there any difference in the cell morphologies of the two algae strains (Fig. 3b–d, f–h). However, in comparison with the non-GIB-treated control, the obvious nuclear and chloroplast for SDEC-11 and clear mitochondrion of SDEC-13 occurred with the addition of phytohormone, which all expressed stronger cell viability. Incorporation of GIB with ADE-KW to culture SDEC-11 is better done in the batch cultivation phase, which yielded a high specific growth rate (0.18 d^−1^). For *Scenedesmus* SDEC-13, the two methods of phytohormone addition had positive effects on the cultures in terms of biomass accumulation and growth rate, in which addition in the seed preparation phase performed better with a specific growth rate of 0.27 d^−1^, compared to 0.18 d^−1^.

From the variations of the cell number (Fig. 1), seed prepared with the hormones significantly increased the cell growths of the two algae strains, especially the maximum cell density achieved on the 10th day (*p* < 0.05). Moreover, it is notable that the GIB phytohormones excited the growth activity of *Scenedesmus* SDEC-13 in ADE-KW to the corresponding level in BG11. The change in cell numbers confirmed the same stimulation by GIB of algae growth as demonstrated for biomass concentration.

The reduction in algae biomass is normal due to the low adaptation of algae in wastewater with complexity [39]. However, compared with GIB-free ADE-KW, a higher activity of the two algae species was observed in ADE-KW inoculated with the algal seed pretreated with phytohormones, where no lag phases occurred. That is to say, priming the algal seed with the phytohormones could mitigate the influence of a different environment, the ADE-KW medium, on algal growth. Moreover, the improvement is more apparent in treated groups of *Scenedesmus* SDEC-13 than *Chlorella* SDEC-11.

### Response of pigment contents to the phytohormones

Photosynthesis in plant and algae cells often undergoes changes in response to environment and phytohormones, and so the changes in the production of pigments involved in photosynthesis were tested (Table 2). *Chlorella* SDEC-11 cultivated in S^+^BG11 groups attained increases in pigment contents of 19% in chl-a and 25% in chl-b. The S^+^ADE-KW group brought enhancement of 33% in chl-a and 26% in chl-b in comparison with the ADE-KW run. The increase in chlorophyll agreed with the appearance of chloroplast in GIB-treated groups compared with thylakoid-like nets in ADE-KW control (Fig. 3c, d). In terms of *Scenedesmus* SDEC-13 grown with ADE-KW, the addition of phytohormones in the seed-preparation phase contributed to significant increases of about 51% in chl-a and 22% in chl-b accumulation in relation to the control. Larger space occupied by chloroplast and more clear thylakoid in it confirmed the increase of GIB on chlorophyll of SDEC-13. The results obtained suggested that stimulation by the GIB phytohormones of photosynthesis and photosynthetic pigment content in algae cells by pretreating the algae seed with GIB allowed the adverse effects of the ADE-KW environment to be endured, while preventing chlorophyll degradation and absorbing limitative light intervened by dark color in wastewater.

**Table 2:** Photosynthetic pigment compositions (mg L^−1^) of *Chlorella* SDEC-11 and *Scenedesmus* SDEC-13 in ADE-KW with phytohormones

Run^a^	Chl-a^1^		Chl-b^2^		Caro^3^		Chl-a + b		Chl-a/Chl-b		Caro/Chl-a + b	
	SDEC-11	SDEC-13	SDEC-11	SDEC-13	SDEC-11	SDEC-13	SDEC-11	SDEC-13	SDEC-11	SDEC-13	SDEC-11	SDEC-13
BG11	4.5 ± 0.2	3.6 ± 0.1	2.4 ± 0.1	2.7 ± 0.8	0.9 ± 0.0	0.7 ± 0.0	7.0 ± 0.3	6.3 ± 0.5	1.9 ± 0.0	1.4 ± 0.1	0.1 ± 0.0	0.1 ± 0.0
ADE-KW	1.2 ± 0.0	2.1 ± 0.2	0.7 ± 0.	1.4 ± 0.1	0.6 ± 0.0	0.8 ± 0.1	1.8 ± 0.0	3.4 ± 0.1	2.2 ± 0.1	1.5 ± 0.0	0.3 ± 0.0	0.2 ± 0.0
S^+^ADE-KW	1.6 ± 0.0	3.1 ± 0.1	0.8 ± 0.0	1.7 ± 0.4	0.6 ± 0.2	0.8 ± 0.2	2.5 ± 0.1	4.8 ± 0.3	2.0 ± 0.1	1.9 ± 0.2	0.3 ± 0.1	0.2 ± 0.0
B^+^ADE-KW	1.4 ± 0.1	2.7 ± 0.2	0.9 ± 0.0	1.5 ± 0.1	0.6 ± 0.4	0.9 ± 0.0	2.3 ± 0.1	4.3 ± 0.2	1.5 ± 0.0	1.8 ± 0.2	0.3 ± 0.1	0.2 ± 0.0

^a^Run names indicate the phase at which hormones were added and the culture medium; e.g., B^+^ADE-KW, indicates that phytohormones were added to algae cultured with ADE-KW at the batch cultivation phase

^1^Chlorophyll-a

^2^Chlorophyll-b

^3^Carotenoids

Tatkowska and Buczek studied the effects of IAA and GA_3_ upon chlorophyll levels of *S. quadricauda* and found that, when added separately, all of these phytohormones stimulated chlorophyll content in algae cells significantly compared with the control [30]. Of the five hormones tested by Park et al., IAA and GA_3_ exhibited the highest increments in concentration of chl-a + b, namely 81 and 68%, respectively [40]. Bajguz and Piotrowska-Niczyporuk reported the positive effect of BL chlorophyll in *Chlorella vulgaris* [12]. Piotrowska-Niczyporuk et al. found that exogenously applied GA_3_ could modify the phytotoxicity of heavy metals on *C. vulgaris* and increase photosynthetic pigment accumulation [15]. Obviously GA_3_, IAA, and BL, either individually or through the combination of these substances, had a beneficial effect on photosynthetic apparatus in microalgae cultivated in general medium or some media with stress. Moreover, carotenoids could have an antioxidative role in the screening and trapping of excessive light, which is otherwise absorbed by the chloroplast [41]. The carotenoid contents of the two algae strains treated with GIB were increased in relation to the corresponding control. Hence, the application of the phytohormones in large-scale cultivation of microalgae should play a positive role in helping algae to mitigate the photoinhibition caused by sunlight.

### Cell morphology in response to the phytohormones

At the early stationary stage of growth, the combination of GA_3_, IAA, and BL significantly affected the cell division expressed as the percentage of cells undergoing cell division and the number of daughter cells retained in a single cell (Fig. 2).

For *Chlorella* SDEC-11, the release of two daughter cells was the main mode of division. The B^+^ADE-KW group of SDEC-11 showed superiority in the amount of cells in division and in the cells that are divided into two daughter cells. Although in S^+^ADE-KW there was no significant increment in the percentage of cells in division, the proportion of cells containing more than two daughter cells increased, which was also a reason for the higher cell density. These results are consistent with the variations of cell density as shown in Fig. 1.

Division into more than two daughter cells was usual in *Scenedesmus* SDEC-13 culture. The GIB hormones significantly promoted cell division via increasing the percentage of cells in division (18.46% in S^+^ADE-KW; 22.38% in B^+^ADE-KW) compared with the ADE-KW control (8.05%) (*p* < 0.05). The stimulation of microalgae by phytohormones was also reported by Park et al., where introducing both GA_3_ and IAA at a single addition time increased the percentage of cells in division and the cells containing four daughter cells [40]. Thus, the present study is the first to report that phytohormones affected the division of microalgae in a manner related to the stage of the hormone dose.

In contrast to cell division, the phytohormones did not generate significant effects on the cell size of *Chlorella* SDEC-11. The dry weight of a cell was calculated according to the corresponding biomass concentration, and cell density was influenced to a great degree by the hormones. For SDEC-11, all the groups under GIB treatment had lighter cells than the other groups, which may be caused by the faster speed of cell division. Especially for the S^+^BG11 group, the interval for daughter cell growth before becoming a mother cell might be shortened by the action of GIB hormones, leading to less material concentration in the cell and lower dry weight compared with the control.

On the basis of the ratio of the A and B axes from SDEC-13, phytohormone treatment resulted in a marked change in morphology. Compared with the ADE-KW control, GIB treatment reduced the value of A/B (*p* < 0.05), which showed sphericalization of cells under the microscope. This phenomenon also occurred in our previous research into the effect of diethyl aminoethyl hexanoate (DA-6) on SDEC-13 [13]. Sphericalization could generate cells with larger specific surface areas to absorb nutrients for growth, which is especially necessary for wastewater treatment. The final concentrations in the water (Table 3) demonstrated the importance of the cell’s character, with lower final nutrient concentration in the S^+^ADE-KW group of SDEC-13. Moreover, cells cultured in ADE-KW from the seed pretreated with GIB weighed 122.55 pg cell^−1^, which is much heavier than the cells grown in ADE-KW without any treatment (103.06 pg cell^−1^) (*p* < 0.05) and even slightly heavier than the cells cultivated in BG11. The change of cell mass could be sensitive to growth medium or phytohormone. In terms of growth medium, Polishchuk et al. used an effluent from anaerobic digestion (DE) of excess-activated sludge and artificial sea water medium (ASW) to grow *Nannochloropsis oculata*, finding that cells grown in the DE were almost twice as heavy as the cells grown in the ASW medium [42].

**Table 3:** The final water characteristics of ADE-KW cultivating *Chlorella* SDEC-11 and *Scenedesmus* SDEC-13

Run^a^	TN (mg L^−1^)	NH_3_-N (mg L^−1^)	TP (mg L^−1^)	COD_Cr_ (mg L^−1^)
*Chlorella* SDEC-11				
ADE-KW	120.7 ± 0.1	66.9 ± 1.3	0.34 ± 0.01	216.8 ± 19.1
S^+^ADE-KW	113.3 ± 1.7	54.8 ± 0.7	0.32 ± 0.01	219.7 ± 25.5
B^+^ADE-KW	111.2 ± 1.3	57.0 ± 0.6	0.24 ± 0.01	208.2 ± 12.8
*Scenedesmus* SDEC-13				
ADE-KW	94.3 ± 1.7	61.7 ± 4.2	0.34 ± 0.03	211.2 ± 13.1
S^+^ADE-KW	66.4 ± 3.0	54.3 ± 1.1	0.42 ± 0.01	203.3 ± 17.3
B^+^ADE-KW	89.4 ± 1.7	57.0 ± 0.6	0.29 ± 0.03	201.6 ± 12.3

^a^Run names indicate the phase at which hormones were added, and the culture medium; e.g., B^+^ADE-KW, indicates that phytohormones were added to algae cultured with ADE-KW at the batch cultivation phase

*Scenedesmus* is a pleomorphic strain which changes its morphology to produce unicells and coenobia under various environmental conditions (length of photoperiod, pH, nutrients) and in response to predators [43]. Compared with BG11, algae in ADE-KW had a smaller proportion of four- and eight-cell coenobia and more two-cell coenobia and unicells. Pancha et al. observed the ecomorphic changes of *Scenedesmus* sp. CCNM 1077, which formed more two- and four-cell coenobia with limited nitrate and sequential nitrate removal [33]. Nevertheless, Siver and Trainor reported a unicell dominance in an ammonia-rich environment [44]. This meant that unicells were produced by *Scenedesmus* sp. with sufficient nitrate. GIB added in the seed preparation or batch cultivation phases triggered morphological changes to four- and eight-cell coenobia and decreased the number of two-cell coenobia in *Scenedesmus* SDEC-13 (Fig. 2b). This was consistent with the finding of Prasad that GA_3_ and IAA promoted the formation of four-celled coenobia in *S. obliquus* rather than two-celled colonies [45]. These results suggested that application of GIB could increase the formation of four- and eight-cell coenobia, and the ammonia and organic carbon in ADE-KW could weaken the ecomorphic expression of the coenobia in *Scenedesmus* SDEC-13.

The GIB phytohormones mitigated the inhibition of ADE-KW on algal cell division, which explained the increment of biomass concentration and cell density in the GIB-treated groups. The morphology including cell size and the number of cell in coenobia also fluctuated under the hormone treatment.

GA_3_, together with IAA from GIB, is usually involved in de-repression of plant-countering stress [17], and organism could recall some events depending on the type of primary stimulus and construct a diverse set of defense mechanisms after being primed [27]. The algal activity in B^+^ADE-KW group indicates the positive function of GIB on algae embracing the wastewater, possibly by means of adjusting internal phytohormone system in algal cell and making chloroplast and nucleus resistant to an unusual environment including imbalance in nutrients, bacterial infection, and dark color. The promotions on chlorophyll, division, and cell morphology of algae in S^+^ADE-KW suggested that the seed prepared with the phytohormones recalls the stimulation of GIB, followed by readily coping with ADE-KW. No lag phase in S^+^ADE-KW also exhibited the system in algal cell being in ready state for confronting some stress or different condition, after the priming process.

### Biomass compositions in response to the phytohormones

**Fig. 4:**
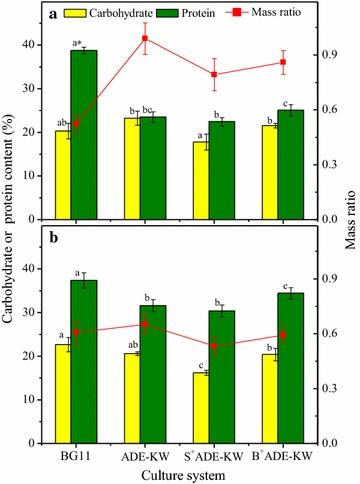
Carbohydrate and protein accumulations of (**a**) *Chlorella* SDEC-11 and (**b**) *Scenedesmus* SDEC-13 cultivated under phytohormones. Mass ratio is the ratio of carbohydrate and protein. The run names on the abscissas indicate the phase at which hormones were added and the culture medium; e.g., B^+^ADE-KW, indicates that phytohormones were added to algae cultured with ADE-KW at the batch cultivation phase. *Data in the same parameter marked with different letters are significantly different by Duncan’s test at *p* < 0.05

**Fig. 5:**
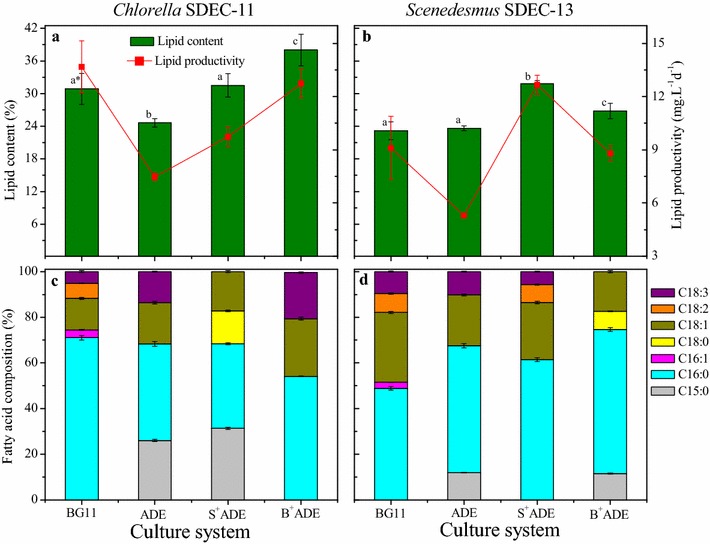
Lipid accumulation (**a**, **b**) and fatty acid profile (**c**, **d**) of *Chlorella* SDEC-11 and *Scenedesmus* SDEC-13 cultivated in ADE-KW under phytohormones. The run names on the abscissa indicate the phase at which hormones were added and the culture medium; e.g., B^+^ADE-KW indicates that phytohormones were added to algae cultured with ADE-KW at the batch cultivation phase. *Data in the same parameter marked with different letters are significantly different by Duncan’s test at *p* < 0.05

Lipid in algae is the most promising component for biofuel production; however, to maximize economic and environmental benefits while minimizing waste and pollution, exploitation of diversity and synthetic biology for the production of algal biofuels is needed [46]. Usually protein and carbohydrate species, the nonlipid fractions, have the potential to produce animal feed, biogas through anaerobic digestion, and so on [47]. Thus, the characteristics of carbohydrate, protein, and lipid in *Chlorella* SDEC-11 and *Scenedesmus* SDEC-13 are evaluated and shown in Figs. 4 and 5 for different culture media and phytohormones.

#### Protein and carbohydrate

In general, protein contents are well correlated with the metabolic activity in the cells [48]. In other words, the accumulation of proteins occurs when the microalgae cells display high metabolic and mitotic activities. The decreased protein content compared to that in BG11 (*p* < 0.05) indicated that the ADE-KW medium was a nonideal environment, as was already indicated by biomass and chlorophyll observations. This environment led to more energy source accumulation for algae self-protection, as Singh et al. concluded that protein content in plants was an important indicator of reversible and irreversible changes in metabolism, a known response to a wide variety of stressors [49]. Another obvious feature of these results was the mass ratio of carbohydrate to protein, which in ADE-KW was higher than that in BG11 for the two algae. The apparent difference in mass ratios of SDEC-11 between ADE-KW and BG11 also suggested a weaker adaptation of *Chorella* SDEC-11 than that of *Scenedesmus* SDEC-13, with a slightly increased mass ratio in ADE-KW. Nevertheless, GIB played a previously unconsidered role in improving the protein activity in the two algae species tested. The protein contents of all groups treated with the phytohormones attained levels that were a little higher than those of the corresponding control without treatment. This same phenomenon occurred in the case of the two algal strains when they were treated by DA-6. No appreciable difference between protein contents in SDEC-11 and SDEC-13 was observed as response to phytohormones, which is different from the noticeable promotion on other algae types reported in the literature [11–13, 15, 39, 49] (Table 4).

**Table 4:** Biomass compositions of microalgae under different phytohormone treatments

Algal species	Phytohormone	Protein content		Carbohydrate content		Reference
		Treated	Control	Treated	Control	
*C. vulgaris*	GA_3_	12.4 × 10^−8^ mg cell^−1^	9.5 × 10^−8^ mg cell^−1^	4.8 × 10^−8^ mg cell^−1^	3.2 × 10^−8^ mg cell^−1^	[11]
*C. vulgaris*	BL and kinetin	390 fg cell^−1^	100 fg cell^−1^	350 fg cell^−1^	60 fg cell^−1^	[12]
*C. vulgaris*	Cytokinins	38 × 10^−8^ µg cell^−1^	18 × 10^−8^ µg cell^−1^	3.8 × 10^−8^ µg cell^−1^	1.1 × 10^−8^ µg cell^−1^	[15]
SDEC-11	DA-6	39%	39%	17%	14%	[13]
SDEC-13	DA-6	37%	39%	15%	13%	[13]
*C. reinhardtii*	1-triacontanol	44% above the control		No difference		[39]
*C. vulgaris*	Indomethacin	43 × 10^−8^ µg cell^−1^	30 × 10^−8^ µg cell^−1^	5.2 × 10^−8^ µg cell^−1^	2.7 × 10^−8^ µg cell^−1^	[49]
SDEC-11	GIB	22/25%^a^	24%^b^	17/22%^a^	23%^b^	This study
SDEC-13	GIB	30/34%^a^	32%^b^	16/20%^a^	21%^b^	This study

^a^The data in this formation present that the former came from S^+^ADE-KW group and the latter from B^+^ADE-KW group

^b^The data come from the ADE-KW control

Unlike protein, the accumulation of carbohydrate in algae was disturbed slightly by ADE-KW and the carbohydrate content decreased to some degree in groups treated with the phytohormones. The reduction was opposite to the phenomenon of higher carbohydrate content under the treatment of phytohormones observed by Falkowska et al. [11], Bajguz and Piotrowska-Niczyporuk [12], Piotrowska et al. [50], and Piotrowska-Niczyporuk et al. [15] (Table 4), which might result from the improved photosynthesis activity. However, Yu et al. also found that gibberellin treatment reduced the content of glucose and promoted the utilization of glucose (carbon source) for conversion into other materials [51]. Compared with cells in ADE-KW, more mitochondria that appeared in cells of SDEC-13 treated by GIB also suggested a possible higher consumption of primary energy materials, such as glucose and starch. There might be another reason that the phytohormone could change the metabolic pathways of carbon. According to the biosynthetic routine of biochemical components in microalgae proposed by Lv et al., diminishment in carbohydrate might be attributed to the transformation of most glyceraldehyde-3-phosphates (G3P), the intermediate product from photosynthesis, into lipid [52].

No linear correlation appeared between carbohydrate content and cell size under the impact of hormones, as shown in the previous research on the effects of DA-6 on SDEC-11 [13]. This might be caused by the effect of GIB on the intracellular carbohydrate synthesis pathway of the strains.

#### Lipid and fatty acid profiles

The effects of the GIB phytohormones on lipid accumulation in SDEC-11 and SDEC-13 cultivated in ADE-KW are evaluated and illustrated in Fig. 5. Considering the effects of growth medium on lipid accumulation, a reduction was observed for SDEC-11 in ADE-KW, and there was no difference between the two media for SDEC-13. However, significant differences (*p* < 0.05) were found for lipid accumulation in the two algal strains between groups with GIB treatment and the corresponding control groups. For lipid content of SDEC-13, all the treatments with the phytohormones exhibited about 130% increase over the control. It is worth noting that the lipid content of SDEC-11 in B^+^ADE-KW reached up to 38%, enhanced by 1.52 times the value of 25% for the GIB-free control, which was visually exhibited by big lipid droplets in ultrastructural cells of B^+^ADE-KW group (Fig. 3a, d). The increase in lipid content balanced the decrease in carbohydrate content, due to the competition for G3P. In previous studies, the auxins appeared not to stimulate significant changes in lipid content [53, 54], but gibberellins promoted a statistical difference in lipid accumulation [50]. It may be GA_3_ in the GIB hormones that worked on lipid content in the two tested algae strains.

Lipid productivity, which combines lipid content and biomass productivity, was decreased by the wastewater through lowering of biomass or lipid accumulation, but all the groups treated with the phytohormones showed superior lipid productivity (*p* < 0.05). Because of the high lipid content, algae SDEC-11 in B^+^ADE-KW group yielded 12.73 mg L^−1^ d^−1^ in lipid productivity with relatively low biomass concentration (0.33 g L^−1^), which was 70% higher than 7.48 mg L^−1^ d^−1^ achieved in ADE-KW control and almost equal to 13.67 mg L^−1^ d^−1^ in BG11. The superiority of GIB treatment with SDEC-13 was more obvious due to the stimulation of both algal growth and lipid biosynthesis. Especially for SDEC-13 in the S^+^ADE-KW group with 12.67 mg L^−1^ d^−1^ in lipid productivity, the treatment increased the lipid productivity by 139% compared to 5.30 mg L^−1^ d^−1^ in ADE-KW and was even higher than that in BG11 (9.10 mg L^−1^ d^−1^).

The fatty acid (FA) profiles of microalgae cultivated in BG11 and ADE-KW media are shown in Fig. 5. The saturated fatty acids were composed of pentadecanoic acid (C15:0), palmitic acid (C16:0), and stearic acid (C18:0). Palmitoleic acid (C16:1) is one of the members of monounsaturated FAs (MUFA), with C18:1 dominating MUFA. Linoleic acid (C18:2) and linolenic acid (C18:3) are polyunsaturated FAs (PUFA). The obvious difference between FA compositions in BG11 and ADE-KW was found in C15:0 and C18:2. C15:0 was present but there was no C18:2 in the two algae strains grown in ADE-KW media, quite the opposite of the result in BG11 media. Treatments of SDEC-11 in S^+^ADE-KW and SDEC-13 in B^+^ADE-KW recorded 21 and 22% increases in saturated fatty acid (SFA) content, respectively, relative to the corresponding values in ADE-KW control; introducing GIB to SDEC-11 during the batch cultivation and to SDEC-13 during the seed preparation decreased the SFA proportion. The influence of the phytohormones on fatty acid composition exhibited a complex difference depending on the dosing phase and algal species. As regards the complicated impacts of plant hormones on FA, Jusoh et al. also found that the operating time of IAA on *C. vulgaris* (UMT-M1) significantly affected the oil accumulation, fatty acid compositions, and gene expression [55].

Differences were exhibited in the influence of GIB on FA composition between the groups with different dosing phases and algal species, which could be further studied in subsequent research. It is encouraging that the GIB phytohormones could make a beneficial change in the lipid biosynthesis pathway.

### The possible mechanism of phytohormone’s influence on algal activity

GA_3_, IAA, and BL in the GIB phytohormones are important plant growth regulators in multiple developmental processes, including orchestration of cellular homeostasis to cope with variable environmental factors, such as ethylene biosynthesis; cell division and elongation; DNA, RNA, and protein synthesis; photosynthesis; and so on [7]. The positive effects of the three phytohormones on the growth or environmental stress response of microalgae have also been explored by many researchers, and are attendant with promotion of cell division and expansion, DNA and RNA levels, shortening the cell developmental cycle, or stimulation of antioxidant enzyme activities, restoring algae growth, and primary metabolite levels [9, 12, 15, 30, 40]. The GIB hormones used in our study stimulated the algae to deal with the unfavorable conditions in ADE-KW, including high ammonia (105 mg L^−1^), limited phosphorus (1.75 mg L^−1^), extreme imbalance in the ratio of nitrogen and phosphorus (78.95), and even dark color and suspended solids as an obstacle to light transmission. These conditions in ADE-KW decreased chlorophyll biosynthesis and light quality reaching the algae, which then resulted in the reduced percentage of cells undergoing division and even reduced lipid content.

**Fig. 6:**
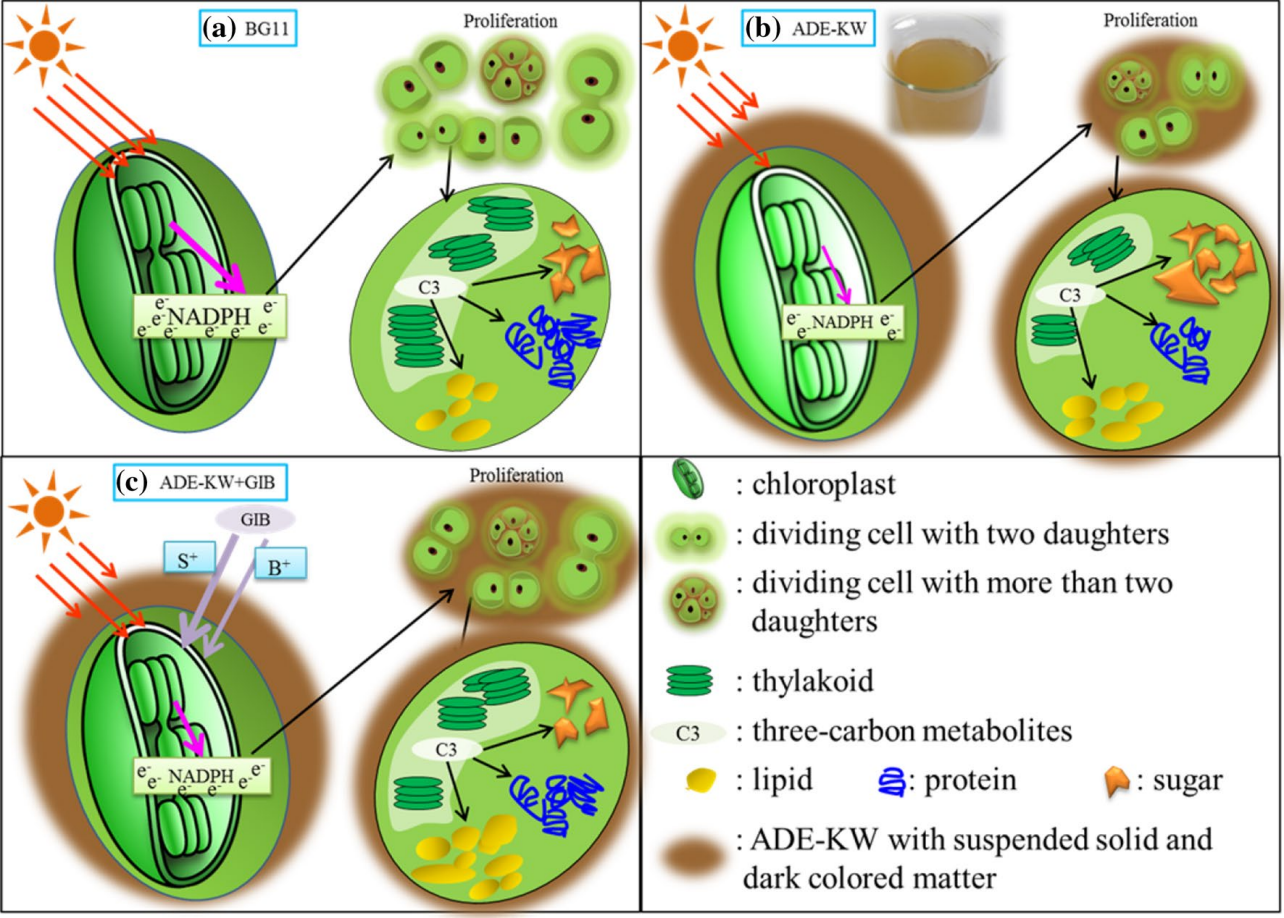
A simplified scheme showing cell division and lipid biosynthesis in microalgae cultivated in **a** BG11, **b** ADE-KW, and **c** ADE-KW with phytohormones addition. The width of the arrows represents the energy flowing into, or the effect on, the material pointed to. *NADPH* nicotinamide adenine dinucleotide phosphate; *ADE-KW* effluent from anaerobic digestion of kitchen waste; *GIB* agricultural phytohormones containing gibberellin, indole-3-acetic acid, and brassinolide; *S* ^*+*^ *ADE-KW* culturing algae in ADE-KW with inoculum pretreated with GIB; *B* ^*+*^ *ADE-KW* introducing GIB into algae in ADE-KW at batch cultivation stage

This stimulation of algae by GIB in ADE-KW might be supported by the regulation of photosynthetic apparatus by the phytohormones. The primary energy source in a microalgae cell is formed in chloroplasts containing chlorophyll, as shown in Fig. 6. Nicotinamide adenine dinucleotide phosphate (NADPH) derived from chlorophyll mainly serves proliferation and cell growth, but lipid biosynthesis is affected when an excess of NADPH appears. Significant improvement of chlorophyll levels by GIB occurred in cells of the two algae strains (increase of 25–40% in total chlorophyll content as shown in Table 2 and improvement of thylakoid or chloroplast as visually revealed in Fig. 3), which could absorb more solar radiation and then more NADPH. This meant more energy was converted into cell proliferation, which ultimately promoted cell division, leading to an increased proportion of SDEC-11 cells containing more than two daughter cells and increased the percentage of SDEC-13 cells undergoing division (Fig. 2). Even lipid accumulation was enhanced when microalgae were confronted with an excess of NADPH following the increment in photosynthetic pigments.

There is a positive correlation between the total chlorophyll content and lipid content for SDEC-11 (*R* ^2^ = 0.45) and, SDEC-13 (*R* ^2^ = 0.92). However such a relationship was not observed when considering data of the two algae species, which might indicate a different energy partitioning between *Chorella* SDEC-11 and *Scenedesmus* SDEC-13. Indeed, the production of NADPH is also related to the condition of thylakoid, in which a functional membrane stack essentially ensures that the photosystem operates efficiently [35, 56]. Apparently for SDEC-11, clear thylakoids in chloroplast were observed after phytohormones treatment, which could be another reason for the higher lipid content in GIB-treated groups. Moreover, Tan and Lee [57] summarized that the generation of NADPH could mainly derive from the metabolism of starch. Compared with ultrastructural cells in ADE-KW control (Fig. 3b), the cells of SDEC-11 (Fig. 3d) in B^+^ADE-KW exhibited fewer starch granules and higher lipid content (Fig. 5). A similar phenomenon between cells of SDEC-13 in control (Fig. 3f) and S^+^ADE-KW (Fig. 3g) was observed.

Carotenoids constitute the main protection against excess light energy and possibly transfer the absorbed radiation [56]. Carotenoid synthesis in algae also increased under the hormone treatment as shown in Table 2, which suggested superfluous light transfer for normal algal growth and the possible excess of NADPH. Over-reduction will occur in the photosynthetic electron transport chain due to richness of NADPH, which could lead to some reactions with highly reduced products, such as lipid synthesis [58]. In addition to carbon supply, de novo FA synthesis also requires a continuous provision of reducing power in the form of NADPH [57]. It may be the efficient stimulation by GIB of chlorophyll in algae that ultimately leads to an increase in lipid accumulation.

The main ingredients in membrane lipid (ML) involve C16:0, C18:0, cis-9-C18:1, cis-9,12-C18:2, and cis-6,9,12-C18:3, and FA in other types are mainly found in storage lipid (SL) [35]. Based on the structural formulae obtained according to the GC-MS retention time, there is no fatty acid in cis-structure present in *Chorella* SDEC-11, and only cis-9-C18:1 and cis-9,12-C18:2 are present in *Scenedesmus* SDEC-13 cultured with BG11. Fatty acids like C16:0 and C18:0 also constitute the main fatty acids in triacylglycerol (TAG), the storage lipid [59], and so, while the value of ML in Additional file 1: Table S2 might not express the membrane activity, the proportion of SL could suggest the percentage of storage lipid. In relation to BG11; the higher SL levels in algae grown in ADE-KW indicated an improvement in storage lipid pathway, which was in agreement with stimulation under a harsh environment of TAG synthesis through production of an excess of NADPH, as visualized in Fig. 6. The GIB phytohormones decreased the content of SL, which might indicate that the cell division occurring in GIB-treated groups, manifesting as increased cell density, consumed NADPH, the feedstock of TAG. Hence, compared with the ADE-KW control, the higher lipid content in GIB-treated groups consisted of more structural lipid or storage lipid formed with saturated fatty acids.

The benefit accrued by the GIB phytohormones to the algae was affected by the biosynthesis and flow direction of NADPH, i.e., the competition of cell proliferation and lipid conversion. NADPH is produced by chloroplasts and sourced from light absorbed by chlorophyll, so the impact of the phytohormones on photosynthesis will be the key to promote simultaneous biomass and lipid accumulation in microalgae.

### Coupling algae-based biofuel production with anaerobic digestion of kitchen waste

**Fig. 7:**
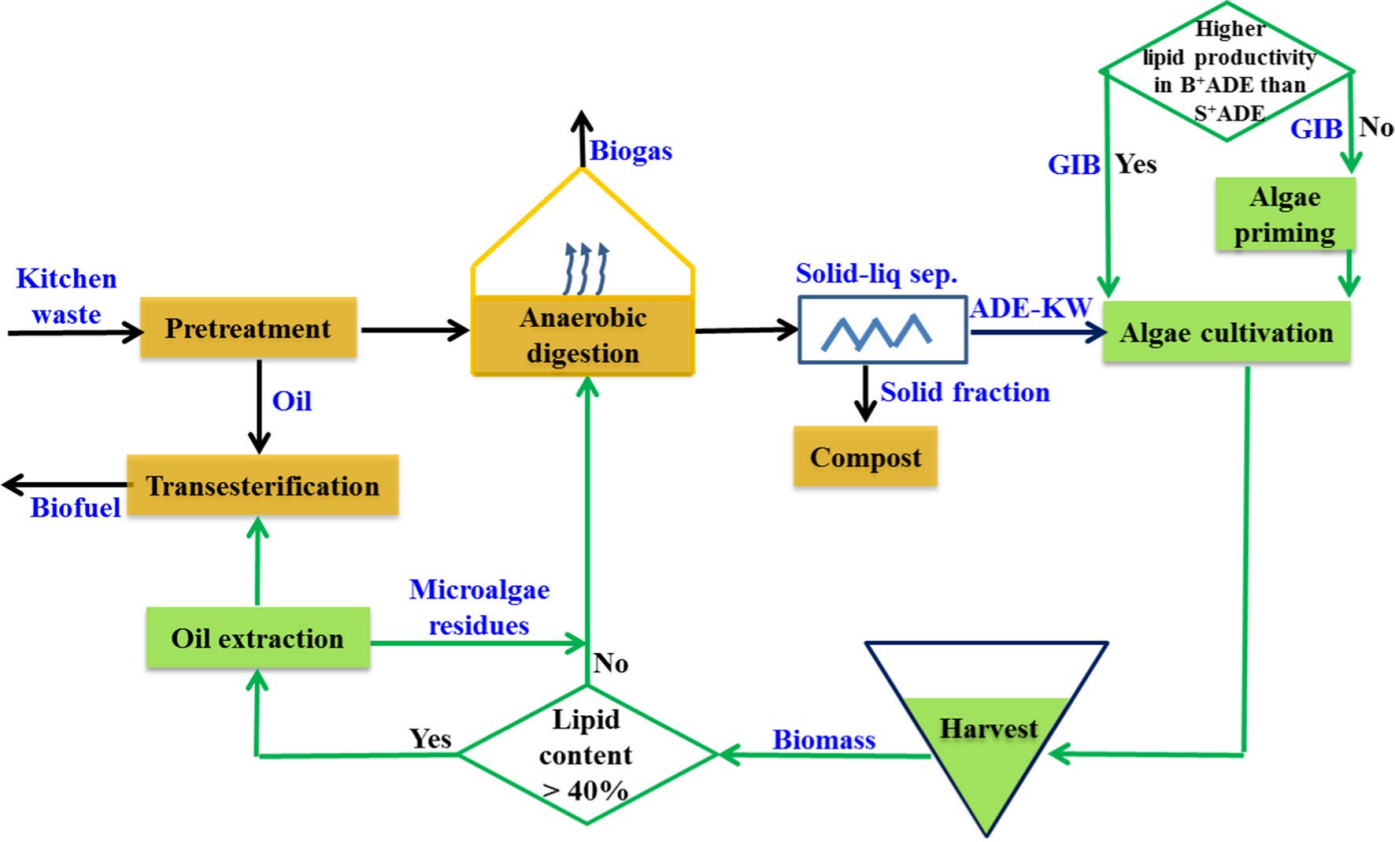
A concept plant for coupling algae-based biofuel production and anaerobic digestion of kitchen waste. ADE-KW: effluent from anaerobic digestion of kitchen waste; *GIB* agricultural phytohormones containing gibberellin, indole-3-acetic, acid and brassinolide; *S* ^*+*^ *ADE-KW* culturing algae in ADE-KW with inoculum pretreated with GIB; *B* ^*+*^ *ADE-KW* introducing GIB into algae in ADE-KW at batch cultivation stage

The above results indicated an improvement by the GIB phytohormones in the adaptability and lipid accumulation of microalgae cultured in effluent from anaerobic digestion of kitchen waste. Table 5 lists the cost of algae biomass production from three culture patterns, BG11, B^+^ADE-KW, and S^+^ADE-KW, without considering the freshwater needed by both dilution of ADE-KW and preparation of BG11. The ADE-KW was used as a zero cost control to estimate the additional investment for nutrients or phytohormones, and the calculation of GIB amount needed by S^+^ADE-KW group was based on the fact that 1 L seed culture was sufficient for 5 L batch cultivation according to the biomass concentrations at the beginning and end of growth period. Apparently, addition of the GIB phytohormones in ADE-KW for algal cultivation is more economic than dosing nutrients in BG11 medium, especially in S^+^ADE-KW cultural pattern where the investment only accounted for a hundredth of that in BG11. The comparison laid a foundation to a concept plant coupling algae-based biofuel production with ADE-KW, as shown in Fig. 7.

**Table 5:** A comparison of costs of algae biomass production from BG11, B^+^ADE-KW, and S^+^ADE-KW, respectively

Algae species	BG11^a^ (CNY ${\text{kg}}_{\text{biomass}}^{{{ - }1}}$)	S^+^ADE-KW (CNY ${\text{kg}}_{\text{biomass}}^{{{ - }1}}$)	B^+^ADE-KW (CNY ${\text{kg}}_{{biomass}}^{{{ - }1}}$)
*Chlorella* SDEC-11	140.3 ± 1.9	17.7 ± 0.7	76.3 ± 2.8
*Scenedesmus* SDEC-13	182.0 ± 15.4	13.5 ± 0.5	75.1 ± 2.6

^a^The cost of BG11 roots in chemicals needed by the medium

In the plant where the ADE-KW was collected, there are mature, advanced technological operations including biodiesel production from the oil separated from kitchen waste and anaerobic digestion of kitchen waste (Fig. 7). This infrastructure could benefit the conversion of the ‘microalgae biomass to biodiesel or biogas,’ Based on the results in the laboratory, the stage for introduction of GIB to algae was set between algae priming and the beginning of algae cultivation, as shown in Fig. 7. Microalgae are then grown in effluent from anaerobic digestion until harvest. The harvested biomass may be sent in two directions, assessed based on lipid content, with a critical point of 40%, according to the report of Sialve et al. [21]. Biomass containing more than 40% lipid enters the transesterification process after oil extraction, and the residues would be anaerobically digested. Chisti discussed the treatment of the microalgae residues after biodiesel production, highlighting its potential to recover biogas and then offset a part of the energy demands of these processes [60]. Based on energy balance and energetic recovery of cell biomass, it is better to transfer the biomass with less than 40% lipid content directly into the anaerobic digestion process with the kitchen waste, which could also improve the methane yield by a reduction of the carbon-to-nitrogen ratio in the digester. The enhancement in the methane yield is normal in co-digestion of algae with other digester feedstocks, because the high nitrogen content in algae could improve the digestibility of carbon-rich substrate [61], like kitchen waste with high ratio of carbon to nitrogen [62].

The concept plant we have proposed and shown in Fig. 7 combines algae-based biofuel production with ADE-KW treatment and forms a cycle where almost no nitrogen or phosphorus is lost. Ajeej et al. proposed that coupling wastewater treatment with the microalgae culture was a promising avenue toward the production of renewable biodiesel or biogas [61]. Moreover, the concept plant based on a kitchen waste treatment plant takes full advantage of the existing transesterification and anaerobic digestion infrastructure, which could mitigate some environmental and economic burdens. This will be an environment-friendly project.

## Conclusions

The study tried to stimulate accumulation of biomass and biocompounds in algae grown in ADE-KW with agricultural phytohormones (GIB) added at different algal growth stages. Regarding cellular ultrastructure under ADE-KW culture, the damage was most obviously observed in cells of SDEC-11 including disintegrated membrane system and formation of vacuoles, while the interference in cells of SDEC-13 just exhibited a decrease in the space between chloroplast and the increase in the size of starch granule. Improvements in terms of biomass concentration, cell density, and photosynthetic pigments of two algae strains could be found in all groups treated by GIB. These characteristics were also exhibited with ultrastructure including stronger chloroplast and cell nucleus. The results were accompanied by variations of cell morphology and cell weight, which were all related to the stages of GIB addition, the growth medium, and the algae species. The accumulations of carbohydrate and lipid differed with regard to different GIB dosing stages and culture media, and there was a positive response to GIB addition in two algae strains cultured with ADE-KW. These effects might have resulted from the synthesis and consumption of G3P and NADPH being influenced by the hormones. It is notable that lipid productivity of SDEC-13 in S^+^ADE-KW increased to 12.67 ± 0.54 mg L^−1^ d^−1^ from 5.30 ± 0.11 L^−1^ d^−1^ under the ADE-KW control. Priming of algal seed with hormones provides an option for improving algal activity in a nonideal environment. Addition of agricultural phytohormones could be a beneficial strategy for promoting the accumulations of biomass and biocompounds in algae cultured with wastewater. Based on these findings, a concept plant for coupling algae-based biofuel production and anaerobic digestion of kitchen waste can be established.

## Additional file

**Additional file 1: Table S1.** Comparison of effluent from anaerobic digestion of different digester feedstocks. **Table S2.** Fatty acid levels (% of total FAME) in *Chlorella* SDEC-11 and *Scenedesmus* SDEC-13.

## Abbreviations

ADE-KW: effluent from anaerobic digestion of kitchen waste
GA_3_: gibberellin
IAA: indole-3-acetic acid
BL: brassinolide
DA-6: diethyl aminoethyl hexanoate
GIB: agricultural phytohormones containing gibberellin, indole-3-acetic acid, and brassinolide
S^+^ADE-KW: culturing algae in ADE-KW with inoculum pretreated with GIB
B^+^ADE-KW: introducing GIB into algae in ADE-KW at batch cultivation stage
FA: fatty acid
MUFA: monounsaturated fatty acid
PUFA: polyunsaturated fatty acid
ML: main fatty acids in membrane lipid
SL: other fatty acids mainly in storage lipid
SFA: saturated fatty acid
G3P: glyceraldehyde-3-phosphate
NADPH: nicotinamide adenine dinucleotide phosphate
TAG: triacylglycerol
