# Supplementary material for: Toward facilitating microalgae cope with effluent from anaerobic digestion of kitchen waste: the art of agricultural phytohormones
Source: Biotechnol Biofuels. 2017 Mar 24;10:76. doi: 10.1186/s13068-017-0759-3 (PMC5366163; doi:10.1186/s13068-017-0759-3)
Supplement: Supplementary file 1 — Additional file 1: Table S1. Comparison of effluent from anaerobic digestion of different digester feedstocks. Table S2. Fatty acid levels (% of total FAME) in Chlorella SDEC-11 and Scenedesmus SDEC-13. [file 13068_2017_759_MOESM1_ESM.docx]

**Table S1** Comparison of effluent from anaerobic digestion of different digester feedstocks.

|  | COD_Cr_ (mg·L^−1^) | TP (mg·L^−1^) | TN (mg·L^−1^) | Digester feedstock | Reference |
| --- | --- | --- | --- | --- | --- |
| 1 | 2661 ± 75 | 381 ± 6 | 2667 ± 30 | municipal wastewater | 2 |
| 2 | 1042 | 75 | 1220 | livestock waste | 9 |
| 3 | 5923 | 47.8 | 2370 | food wastewater | 10 |
| 4 | - | 105.8 ± 1* | 1447 ± 46* | municipal wastewater | 11 |
| 5 | 67.63 ± 0.99 | 86.01 ± 34* | 361.35 ± 61.01* | dry algae biomass | 12 |
| 6 | 2100 | 140 | 300 | mixture of dairy manure and food wastes | 13 |
| 7 | 6096 ± 34 | 20 ± 6 | 2016 ± 17 | kitchen waste | This study |

* Values for PO_4_-P and NH_3_-N.

**Table S2** Fatty acid levels (% of total FAME) in *Chlorella* SDEC-11 and *Scenedesmus* SDEC-13.

| Run^a^ | SFA | MUFA | PUFA | ML | SL |
| --- | --- | --- | --- | --- | --- |
| *Chlorella* SDEC-11 | |  |  |  |  |
| BG11 | 71.03 ± 1.05 | 17.25 ± 0.43 | 11.71 ± 0.30 | 71.03 ± 1.05 | 28.97 ± 1.05 |
| ADE-KW | 68.28 ± 1.14 | 18.15 ± 0.58 | 13.57 ± 0.42 | 42.33 ± 1.04 | 57.77 ± 1.04 |
| S^+^ADE-KW | 82.84 ± 0.44 | 17.16 ± 0.41 | 0 | 51.47 ± 0.27 | 48.53 ± 0.27 |
| B^+^ADE-KW | 54.29 ± 0.22 | 25.36 ± 0.55 | 20.35 ± 0.01 | 54.12 ± 0.25 | 45.88 ± 0.25 |
| *Scenedesmus* SDEC-13 | |  |  |  |  |
| BG11 | 48.85 ± 0.89 | 33.27 ± 0.24 | 17.88 ± 0.53 | 87.65 ± 0.65 | 12.35 ± 0.65 |
| ADE-KW | 67.51 ± 0.91 | 22.29 ± 0.22 | 10.20 ± 0.43 | 55.55 ± 0.87 | 44.45 ± 0.87 |
| S^+^ADE-KW | 61.44 ± 0.83 | 25.14 ± 0.60 | 13.46 ± 0.45 | 61.44 ± 0.83 | 38.56 ± 0.83 |
| B^+^ADE-KW | 82.65 ± 0.78 | 17.35 ± 0.47 | 0 | 71.16 ± 0.61 | 28.84 ± 0.61 |

a Run names indicate the phase at which hormones were added and the culture medium; *e.g*. B^+^ADE-KW indicates that phytohormones were added to algae cultured with ADE-KW at the batch cultivation phase.

SFA: saturated fatty acids (C15:0, C16:0, C18:0); MUFA: monounsaturated fatty acids (C16:1, C18:1); PUFA: polyunsaturated fatty acids (C18:2, C18:3); ML: main fatty acids in membrane lipid; SL: other fatty acids mainly in storage lipid.
